# Autoimmune Disease pH and Temperature

**DOI:** 10.14740/jocmr1839w

**Published:** 2014-05-22

**Authors:** Kevin Carlin

**Affiliations:** Central Texas Veterans Health Care System, Temple, TX, USA. Email: Kevin.Carlin@VA.GOV

## To the Editor

Perhaps parts of the immune system are pH and temperature sensitive. If true, there could be a variance in pH and/or temperature under certain conditions that could impact the immune system and vice versa. Possibly the illusive etiology of autoimmune disease is in part due to pH and/or temperature changes.

A majority would probably agree the human immune system is designed to destroy foreign invasion such as an infection and/or for removal of nonviable tissue such as that damaged by trauma. Obviously, both of the previous situations would be more likely to involve at least a local environment trending more acidic.

Also most would agree autoimmune disease is when there is an error in the immune system and the system begins to attack and destroy viable tissue. Thus could the etiology of autoimmune disease be a repair system gone awry possibly because of a pH and/or temperature change? If cancer is the repair system gone awry in regard to overproduction of replacement cells, then perhaps autoimmune disease is the repair system gone awry in regard to the damaged cell removal causing disease possibly because of a pH and/or temperature change.

When there is cell damage inflammation develops as certain cells infiltrate the area and certain mediators accumulate that are also part of the immune system. Autoantibodies have been noted to be present following trauma with some suspecting this is a mechanism to remove cellular debris. However, autoantibody presence suggests a possible autoimmune disease in the future but is not diagnostic, since many normal individuals have mild levels. For example, with diabetes mellitus type I, there can be an autoantibody present for years (or even a life time) without any overt disease ever developing. In short, autoimmunity may follow tissue damage with low levels of antibodies but not necessarily actual autoimmune disease with destruction of viable tissue. So this is similar to other situations where something could occur, but does not have to occur, or rather something is more likely to occur, but is still not a certainty. An unknown second step probably has to occur, could that second step involve a pH and/or temperature change is the question.

If an individual develops an autoimmune disease, they are more likely to develop a second autoimmune disease than are other patients. Autoimmune diseases seem to run in families as well. There appears to be a genetic susceptibility. Some say autoimmune diseases are multigenic, heterogeneous in genetic basis, with identical twins mixed on penetrance but usually higher than siblings and much higher than unrelated populations. If there is a pH aspect to the immune system, perhaps this involves the cellular pumps, channels, transporters and isoenzyme levels. This could account for a familial predisposition yet be multigenic, since there are so many different cellular pumps, channels, transporters and isoenzymes. There are multiples of each of these, so a particular combination could make an individual susceptible and this would be genetic. The cellular pumps, channels, transporters and isoenzymes could also respond to environmental stress changing to some degree accounting for differences in identical twins.

The medications procainamide and hydralazine have been known for decades to induce autoimmune disease, specifically lupus. Procainamide impacts sodium channels while hydralazine impacts the calcium activated potassium channels and so both could impact pH levels. Interestingly, some compliment activation [1] and IgG binding [2] are more reactive in an acidic environment. In addition, some interleukins seem to prefer an acidic environment [3]. But there is also a possibility extreme levels of alkalinity can set the immune system into action as well, at the very least through cell destruction.

Strong ions such as iodine in the thyroid could conceivably impact the cellular pumps, channels, transporters and isoenzymes. Indeed that is possibly what we see. As the level of intrathyroidal iodine increases, so does the incident of Hashimoto’s thyroiditis [4], one of the most common autoimmune diseases. However, not everyone develops Hashimoto’s thyroiditis with higher iodine, just some people. Could those with a certain mix of cellular pumps, channels, transporters and isoenzymes be the ones that are more likely to develop the Hashimoto’s thyroiditis? Also some cytokines such as interferon can induce Hashimoto’s thyroiditis. Could interferon possibly be impacting the cellular pumps, channels, transporters and isoenzymes? yes, it is known to impact the potassium channels for example [5]. Finally, if pH changes enough there could be a charge generated which could impact movement possibly even for T and B cells.

Another interesting aspect about autoimmune diseases is they are in general significantly more common usually in females than males. Women have increased antibody production compared to men in response to infection, vaccination and trauma. This could be due to pH differences. Testosterone is converted into dihydrotestosterone to be more effective by 5-alpha reductase (1.3.99.5) type II to cause for example male balding, the optimum pH is 5.5 [6]. Testosterone can also be converted into estrogen products by aromatase. Aromatase (1.14.14.1) has an optimum pH of 7.4 - 10.4 [7] to convert androgens into estrogens. Furthermore, in rabbit uterus tissue, 17-beta hydroxysteroid dehydrogenase (1.1.1.51) has optimum pH of 9.5 - 10.5 [8] changing estrodiol into estrone while at pH 5.5 - 6.5 the change from estrone into estrodiol occurs optimally. So there could be a pH preference at work. Specific estrogens may stimulate one type of pumps while specific androgens stimulate another. Indeed estrodiol inhibits voltage-gated potassium channels while testosterone has far less impact on that particular channel [9].

With ovulation females have a significant measurable temperature rise. Hypothermia treatment experts (for example cooling for cardiac surgery) have known for years there is a rise in pH toward alkalinity with cooling [10], so perhaps there is a decrease toward acidity with an increase in temperature. Indeed, the immune system may prefer a temperature increase. We have known for almost 50 years that cancer and areas of inflammation are warmer, rheumatoid joints have been shown to be warmer, and surgery (organized trauma) induces temperature increase that resolves with healing. Could temperature and pH be involved with the immune system response in some cases?

The mouse thymus with external heating of the animals has been found to decrease the percentage of immature double positive cells and an increase in the percentage of mature cells (no more cells rather just a change in the mix) [11]. Perhaps this reflects what good cooks have known for years. If one cooks too fast at too high a temperature, the meal may not be optimum. Perhaps thymus processed T cells are consequently impacted. In short, what might be good for the immediate situation could have a negative long term impact.

Brown fat is well known to be involved in thermogenesis. The fact that brown fat is located around the thymus may not be a random act. It also probably is not a random act that the following possess pyrogenic activity: IL-1 alpha and beta, TNF-alpha and beta, interferon alpha and gamma, IL-2, and IL-6 [12]. Also T helper cells secrete more of some cytokines at higher temperatures [12]. Why would so many immune system products induce an increase in temperature unless it was to the immune system’s advantage?

Chemical reactions need energy/heat/electrical charge or give off energy/heat/electrical charge, an excess that does not dissipate fast enough may set off the immune system. Perhaps we need to think of energy/temperature like water. It can come as a drip at times, but these add up to lots of water potentially which can dissipate or accumulate in puddles. Energy that failed to dissipate and collected in puddles could alter the immediate environment through heat. Thus anything causing an accumulation or lack of dissipation could impact the immediate environment. With chemical reactions continuously occurring in the human body in every single cell how could this not potentially be a factor at least on occasion?

If heat by itself is an instigator of the immune system under certain circumstances, then an exothermic reaction in the small intestine could impact the head of pancreases which is covered on three sides by the small intestine. This by itself or in conjunction with fever from another cause could raise temperature and set off a cascade. However, virus would not be found in the beta islet cells of the pancreas. For decades researchers have looked for evidence of a virus in the type I diabetic pancreas without success. In addition, the overall pancreas could also have a pH change with the production of so much bicarb for the small intestines. Thus, sometimes it maybe temperature, while other times pH, and others a combination of the two that could set off the immune system.

Since shortly after WWII we have known corticosteroids decrease the size of the thymus. The Na/H exchanger activity level is known to be impacted by glucocorticoids. Glucocorticoids increase the Na-H exchange and decrease the Na gradient [13]. This could cause a pH change. Perhaps thymus involution is at least in part a pH change.

All of the above could just be coincidental, but an actuary would say unlikely. The immune system seems to be sensitive to pH and temperature changes as well as vice versa.
